# Comparison of Microbial Communities in the Sediments and Water Columns of Frozen Cryoconite Holes in the McMurdo Dry Valleys, Antarctica

**DOI:** 10.3389/fmicb.2019.00065

**Published:** 2019-02-04

**Authors:** Pacifica Sommers, John L. Darcy, Dorota L. Porazinska, Eli M. S. Gendron, Andrew G. Fountain, Felix Zamora, Kim Vincent, Kaelin M. Cawley, Adam J. Solon, Lara Vimercati, Jenna Ryder, Steven K. Schmidt

**Affiliations:** ^1^Ecology and Evolutionary Biology, University of Colorado Boulder, Boulder, CO, United States; ^2^Department of Botany, University of Hawai‘i Mānoa, Honolulu, HI, United States; ^3^Department of Entomology and Nematology, Institute of Food and Agricultural Sciences, University of Florida, Gainesville, FL, United States; ^4^Geology Department, Portland State University, Portland, OR, United States; ^5^National Ecological Observatory Network Operated by Battelle, Boulder, CO, United States; ^6^Institute of Arctic and Alpine Research, University of Colorado Boulder, Boulder, CO, United States

**Keywords:** Antarctica, cryoconite, niche partitioning, extremophile, bacteria, ciliate

## Abstract

Although cryoconite holes, sediment-filled melt holes on glacier surfaces, appear small and homogenous, their microbial inhabitants may be spatially partitioned. This partitioning could be particularly important for maintaining biodiversity in holes that remain isolated for many years, such as in Antarctica. We hypothesized that cryoconite holes with greater species richness and biomass should exhibit greater partitioning between the sediments and water, promoting greater biodiversity through spatial niche partitioning. We tested this hypothesis by sampling frozen cryoconite holes along a gradient of biomass and biodiversity in the Taylor Valley, Antarctica, where ice-lidded cryoconite holes are a ubiquitous feature of glaciers. We extracted DNA and chlorophyll *a* from the sediments and water of these samples to describe biodiversity and quantify proxies for biomass. Contrary to our expectation, we found that cryoconite holes with greater richness and biomass showed less partitioning of phylotypes by the sediments versus the water, perhaps indicating that the probability of sediment microbes being mixed into the water is higher from richer sediments. Another explanation may be that organisms from the water were compressed by freezing down to the sediment layer, leaving primarily relic DNA of dead cells to be detected higher in the frozen water. Further evidence of this explanation is that the dominant sequences unique to water closely matched organisms that do not live in cryoconite holes or the Dry Valleys (e.g., vertebrates); so this cryptic biodiversity could represent unknown microbial animals or DNA from atmospheric deposition of dead biomass in the otherwise low-biomass water. Although we cannot rule out spatial niche partitioning occurring at finer scales or in melted cryoconite holes, we found no evidence of partitioning between the sediments and water in frozen holes. Future work should include more sampling of cryoconite holes at a finer spatial scale, and characterizing the communities of the sediments and water when cryoconite holes are melted and active.

## Introduction

Cryoconite holes are a unique aquatic environment found on glacier surfaces that support active microbial communities capable of playing a significant role in regional nutrient cycles. They start as sediment patches that accumulate on icy surfaces (McIntyre, 1984). Solar radiation heats the sediment more efficiently than the surrounding ice because of its lower albedo. The warmer sediment melts the underlying ice and migrates downward, forming holes of meltwater that deepen quickly to an equilibrium depth, where the rate of deepening is equal to the ablation rate of the ice surface (Gribbon, 1979; Wharton et al., 1985; Fountain et al., 2008). On the glaciers in the McMurdo Dry Valleys of Antarctica, the surface energy balance is such that the ubiquitous water-filled holes quickly freeze over, leaving a subsurface pool of water that can persist for over a decade, melting every austral summer (Fountain et al., 2004; Tranter et al., 2004). However, in warm years, they may also flush with their accumulated carbon and nitrogen into ice-covered lakes and contribute to increased productivity (Bagshaw et al., 2013).

The nitrogen from cryoconite holes is fixed by active microbial communities (Wharton et al., 1985; Christner et al., 2003; Tranter et al., 2004; Foreman et al., 2007; Telling et al., 2014) which exhibit distinct vertical partitioning of at least the ciliate community during summer melt-out (Mieczan et al., 2013). Given the differences in light availability (Bagshaw et al., 2016b), cell densities (Foreman et al., 2007), and differences in nutrients and other physicochemical characteristics (Foreman et al., 2007; Mieczan et al., 2013) between the bottom sediment and the overlaying water, other taxa also are likely to partition between the sediments and water within cryoconite holes. Partitioning at the water-sediment interface can be driven by physical processes such as freeze concentration of impurities (gasses, ions, and particles) as the formation of ice rejects those impurities (as in Wait et al., 2006). It can also be generated by the organisms themselves through movement or population growth (Mieczan, 2010; Mieczan et al., 2013). For species requiring similar resources, but whose favorable locations differ, this can lead to spatial niche partitioning, promoting coexistence and enhancing biodiversity (Levin, 1974; Chesson, 2000; Cardinale, 2011). Not only could distinct spatial niche partitioning at the very coarse scale of sediment and water promote species richness through coexistence, but greater biomass and richness could enhance partitioning by forcing species to specialize their growth in either sediments or water through competitive interactions. Either of those dynamics would lead to more distinctive communities in the sediments and water in richer cryoconite holes than in lower biomass and lower richness holes.

To test whether we observed less overlap in communities between the water and sediments of richer cryoconite holes, we sampled frozen cryoconite holes across three glaciers representing a gradient of phylodiversity (Sommers et al., 2018) and biomass (Porazinska et al., 2004) in the Taylor Valley, Antarctica. Specifically, we employed high-throughput amplicon sequencing to characterize bacterial and microbial eukaryotic communities in each layer and to determine the percentage of phylotypes in the water also detected in the sediments, and vice versa. To verify the gradient, we also determined the richness of phylotypes, and we measured total carbon, chlorophyll *a*, and DNA as proxies for biomass. We expected that the structure of microbial communities would differ between the sediments and water to at least some degree, as they do in other Antarctic aquatic ecosystems (e.g., Archer et al., 2014, 2015). For example, we expected that non-cyanobacterial primary producers such as algae, and motile taxa such as ciliates would be found primarily in the water, while cyanobacteria would form mats in the sediments, associated with less motile grazers such as tardigrades. We also expected that like most aquatic environments, including cryoconite holes on one of our sampled glaciers, the sediments would have greater biomass than the water (Foreman et al., 2007). Despite this difference, we expected that the gradient of biomass across the glaciers would be reflected in both the water and sediments.

## Materials and Methods

### Field Site, Sample Collection, and Processing

The McMurdo Dry Valleys, Antarctica, stretch from the edge of the Antarctic ice sheet to the coast at McMurdo Sound. Our field study was in the Taylor Valley, a ∼40 km long, ∼12 km wide valley between two mountain ranges. The valley floor is dotted with lakes, exposed bedrock, with large expanses of poorly developed soils (Priscu, 1999). A major geologic feature, the Nussbaum Riegel, divides Taylor Valley physically and climatically into two main basins, the warmer (in summer), drier Lake Bonney basin to the west which is adjacent to the ice sheet; and the cooler, wetter Lake Fryxell basin to the east adjacent to the Sound (Fountain et al., 1999, 2010; Barrett et al., 2006). A third, much smaller basin, is formed by Canada Glacier on the north side of the Riegel, which dams the natural drainage to Lake Fryxell, forming Lake Hoare.

The major wind directions are channeled by the mountains that border the valley and can be divided into up-valley or down-valley. In summer the more gentle breezes are up-valley, onshore winds from the Ross Sea. The stronger, particularly in winter, winds are down-valley föhn winds descending from the ice sheet (Nylen et al., 2004; Šabacká et al., 2012). The föhn winds are particularly important because they can transport sediments and biota from the valley floor soils, stream channels, and lakes on to the glaciers.

We sampled cryoconite holes between 7 and 17 November, 2016, while they were frozen, on three different glaciers, Taylor (“Tay”), an outlet glacier of the ice sheet at the head of the valley, Canada (“Can”), about midway down-valley, and Commonwealth (“Com”), at the valley mouth by the McMurdo Sound. We used a SIPRE corer to drill cores 20 (± 10) cm long and 10 cm in diameter. We stored the cores in sterile Whirl-Pak bags (Nasco, Fort Atkison, WI, United States) at −20°C for up to 1 month. In the Crary Laboratory at McMurdo Station, cores were separated into the sediment layer and the overlaying ice. Each component was separately washed with deionized water to melt off the outer layer and potential cross-contamination from the drill, then placed in separate, acid-washed, high-density polyethylene (HDPE) beakers covered with aluminum foil and melted at 4°C for 12–24 h. Final melting took place at room temperature when necessary immediately prior to homogenizing, subsampling, and filtering.

### DNA Extraction and Sequencing

For DNA extractions from the sediment layer, excess water was poured off and 0.3–0.4 g wet sediment was transferred to a PowerSoil DNA Isolation Kit (MoBio Inc., Carlsbad, CA, United States) bead beating tube. For DNA extractions from the water, 50 ml water was poured through a Whatman Nuclepore (GE Healthcare, Pittsburg, PA, United States) 0.2 μm filter, and that filter was transferred to a PowerWater DNA Isolation Kit (MoBio Inc., Carlsbad, CA, United States) bead beating tube. Tubes were frozen at −20°C until the DNA was extracted, up to 1 month later, following the manufacturer’s protocol with one exception. For the final step in eluting the DNA from the spin filter in the water extractions only, we used only half the final solution in order to increase the concentration of DNA because we expected it to be near detection thresholds. For 15 arbitrarily selected samples from the sediments and the water on each glacier (45 sediments and 45 water total), we measured the concentration of extracted DNA using a Qubit fluorometer (Qubit, London, United Kingdom), according to the manufacturer’s instructions. DNA concentrations were back-calculated to be ng per approximate cubic centimeter (g dry sediment or ml water).

Extracted genomic DNA was amplified in triplicate using 16S (515f-806r primers, Caporaso et al., 2012) and 18S (1391f-EukBr primers, Amaral-Zettler et al., 2009; Caporaso et al., 2012) SSU ribosomal gene markers. Amplified DNA was pooled and normalized to equimolar concentrations using SequalPrep Normalization Plate Kits (Invitrogen Corp., Carlsbad, CA, United States), and sequenced using the Illumina MiSeq V2 (2 × 250 bp chemistry) at the BioFrontiers Sequencing Core Facility at the University of Colorado at Boulder. Sequences have been deposited in the NCBI SRA database under project PRJNA480849.

### Biomass and Production

As a measure of past primary production biomass, we measured chlorophyll *a* concentrations in the sediments and the water. For chlorophyll extractions from the water, 50 ml of melted ice was filtered through a Whatman GF/F glass fiber filter (GE Healthcare, Pittsburg, PA, United States). For chlorophyll in the sediments, 5 g sediments were mixed with 50 ml melted ice from the same cryoconite hole, then allowed to settle for at least 5 min before being filtered. We wrapped the filters in aluminum foil and stored them at −20°C for up to 2 months before acetone extraction, which followed the methods of Chiuchiolo (2017). The filters were extracted in 10 ml 90% acetone for 24 h with agitation at 12 and 24 h, then 4 ml was pipetted into a cuvette and fluorescence was measured with a 10-AU Fluorometer (Turner Designs, Sunnydale, CA, United States). Measurements were back-calculated to a chlorophyll concentration based on a regression of known chlorophyll standard solutions, then further transformed for sediment samples to a per kg dry mass equivalent, and to account for the chlorophyll already in that water sample.

### Data Processing and Analysis

Raw reads were de-multiplexed and quality filtered using the QIIME v1.9.1 bioinformatics package (Caporaso et al., 2010a,b), using paired-end sequences that were joined with VSEARCH (Rognes et al., 2016). Bacterial and eukaryotic sequences were separately clustered into OTUs at 97% similarity using UCLUST (Edgar, 2010). Taxonomy was assigned using QIIME’s parallel_assign_taxonomy_blast.py script with the SILVA 128 Ref NR99 database’s taxonomic information (Quast et al., 2013). All mitochondrial and chloroplast OTUs based on this classification were removed from the bacterial data set and all bacterial OTUs were removed from the eukaryotic data set. OTUs that made up at least 1% of the extraction blank sequences were discarded as likely lab contamination, with two bacterial exceptions (both Bukholderiales) that were dominant members of the sediment community, where contaminants are unlikely to dominate, given the DNA concentrations. Common sequences from real samples could show up in the low-DNA blanks from pipette aspiration. Singleton OTUs were removed. The reads per sample were scaled by the g dry sediment or ml of water extracted to ensure more even comparison, and multiplied by 1,000 to maintain rare OTUs above zero before being rounded to nearest integers. Samples were then rarefied to 6,900 bacterial sequences and 5,680 eukaryotic sequences each. Richness of OTUs was calculated as the number of OTUs in the samples after transformation and rarefaction. The taxonomic assignments of the dominant OTUs from each component (water and sediments) on each glacier set were verified with the NCBI non-redundant ribosomal database using BLAST.

Because we collected samples over a gradient of phylodiversity and microbial abundance within cryoconite holes along the Taylor Valley (Porazinska et al., 2004; Sommers et al., 2018), we expected differences among glaciers and between sediments vs. water. We therefore analyzed differences in the OTU richness per cm^3^ with a two-way analysis of variance (ANOVA) including an interaction term, log transforming the data where appropriate. We used Tukey HSD *post hoc* comparisons of groups for more detailed discussion, as implemented in R 3.3.2 (R Core Team, 2016). We took a similar approach to analyzing differences in chlorophyll and DNA.

We calculated the Bray-Curtis pairwise dissimilarity metric (Bray and Curtis, 1957) for all samples using QIIME. We used a principal coordinates analysis (PCoA) implemented in package “ape” (Paradis et al., 2004) in R to visualize the results, and a permutational multivariate analysis of variance (PERMANOVA) implemented in package “vegan” (Oksanen et al., 2018) with *post hoc* pairwise comparisons in “RVAideMemoire” (Hervé,, 2018) to ask how assemblage structures differed among habitats and glaciers.

To compare the overlap in OTUs detected in the sediment and water we calculated the percent of OTUs in each habitat (water or sediments) also detected in the other for that sample. These were also compared with an interactive two-way ANOVA, log transforming data where appropriate.

## Results

### Microbial Assemblages

**FIGURE 1:**
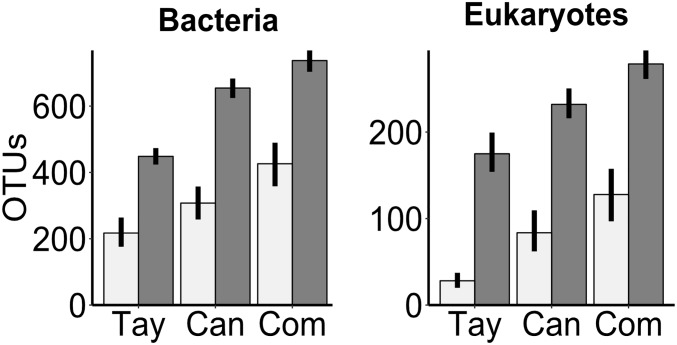
Richness of OTUs of bacteria and microbial eukaryotes in the sediment and water of cryoconite holes across three glaciers along a gradient of diversity. Tay: Taylor Glacier, Can: Canada Glacier, Com: Commonwealth Glacier. Richness in water (light bars) is scaled up to 1 l of water, and richness in sediments (dark bars) to 1 kg dry sediment. Error bars are bootstrapped 95% confidence intervals implemented in “ggplot2” (Wickham, 2009) in R.

As expected, the overall richness of bacterial OTUs (*n*_1_ = *n*_2_ = 74) was 50% or more greater in sediments (scaled to 1 kg dry sediment) than in water (scaled to1 l) (*F* = 136, *P* < 0.001), and this pattern was consistent across all glaciers. Also as expected, Commonwealth Glacier was the richest and Taylor was the most depauperate (*F* = 27, *P* < 0.001) (Figure 1). The richness of eukaryotic OTUs (*n*_1_ = *n*_2_ = 71) was similarly greater in the sediments than the water (*F* = 183, *P* < 0.001), with richness also differing significantly across glaciers (*F* = 34, *P* < 0.001), and a significant interaction between glacier and sample type (water vs. sediment) (*F* = 6.4, *P* = 0.002) (Figure 1).

**FIGURE 2:**
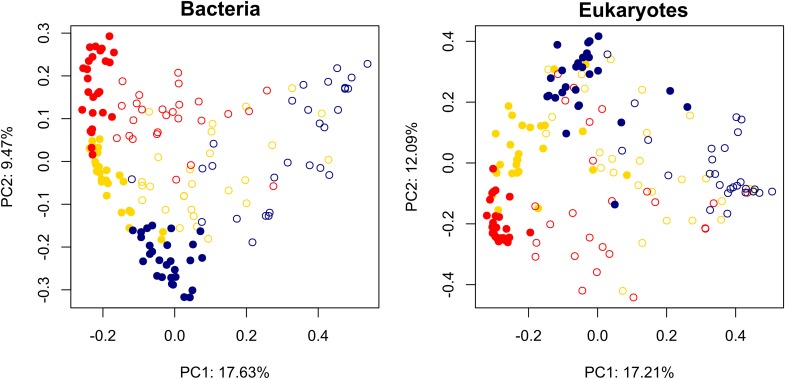
Principal coordinates analysis biplot of Bray-Curtis dissimilarity metric for bacterial and eukaryotic assemblages in cryoconite holes on three glaciers along a gradient of diversity. Solid circles: sediments; open circles: water. Red: Commonwealth Glacier; gold: Canada Glacier; navy: Taylor Glacier.

Also as expected, assemblages of both bacteria and microbial eukaryotes differed significantly between sediments vs. water by glacier (bacteria *F* = 35, *P* = 0.001; eukaryotes *F* = 40, *P* = 0.001; Figure 2). More specifically, *post hoc* comparisons showed that all were significantly distinct from one another (*P* < 0.01 for all). Although dispersion of community distances differed significantly among comparisons both for bacteria (*F* = 19, *P* < 0.001) and eukaryotes (*F* = 26, *P* = 0.043), PERMANOVA is robust to heterogeneous dispersions for balanced designs (Anderson and Walsh, 2013).

**FIGURE 3:**
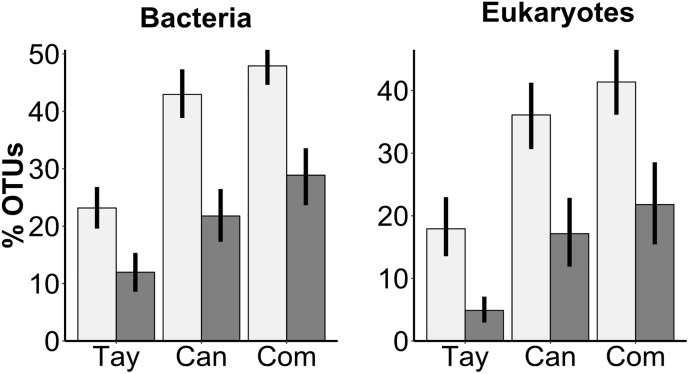
Percent of OTUs found in the water of a given cryoconite hole also found in its sediment (light bars), and OTUs from the sediment also found in that hole’s water (dark bars) for both bacteria and microbial eukaryotes across three glaciers along a gradient of diversity. Tay: Taylor Glacier, Can: Canada Glacier, Com: Commonwealth Glacier. Error bars are bootstrapped 95% confidence intervals implemented in “ggplot2” (Wickham, 2009) in R.

We next determined whether the communities in the water were a subset of the communities in the sediments by calculating what proportion of OTUs from each stratum were also detected in the other. We found that significantly higher percentages of bacterial OTUs detected in the water were also found in the sediments than vice versa (*F* = 100, *P* < 0.001), and that the percentage differed by glacier (*F* = 54, *P* < 0.001), with a significant interaction between sediment-water comparison and glacier (*F* = 3.3, *P* = 0.040) (Figure 3). Specifically, the percentage of overlapping bacterial OTUs in both the sediments (Tukey HSD: Tay-Can: *P* = 0.012; Tay-Com: *P* < 0.001; Can-Com: *P* = 0.16) and water (Tukey HSD: Tay-Can: *P* < 0.001; Tay-Com: *P* < 0.001; Can-Com: *P* = 0.54) were higher on Canada and Commonwealth Glaciers than on Taylor Glacier. The percent of overlapping eukaryotic OTUs also varied between sediments and water (*F* = 62, *P* < 0.001) and by glacier (*F* = 31, *P* < 0.001) (Figure 3).

**FIGURE 4:**
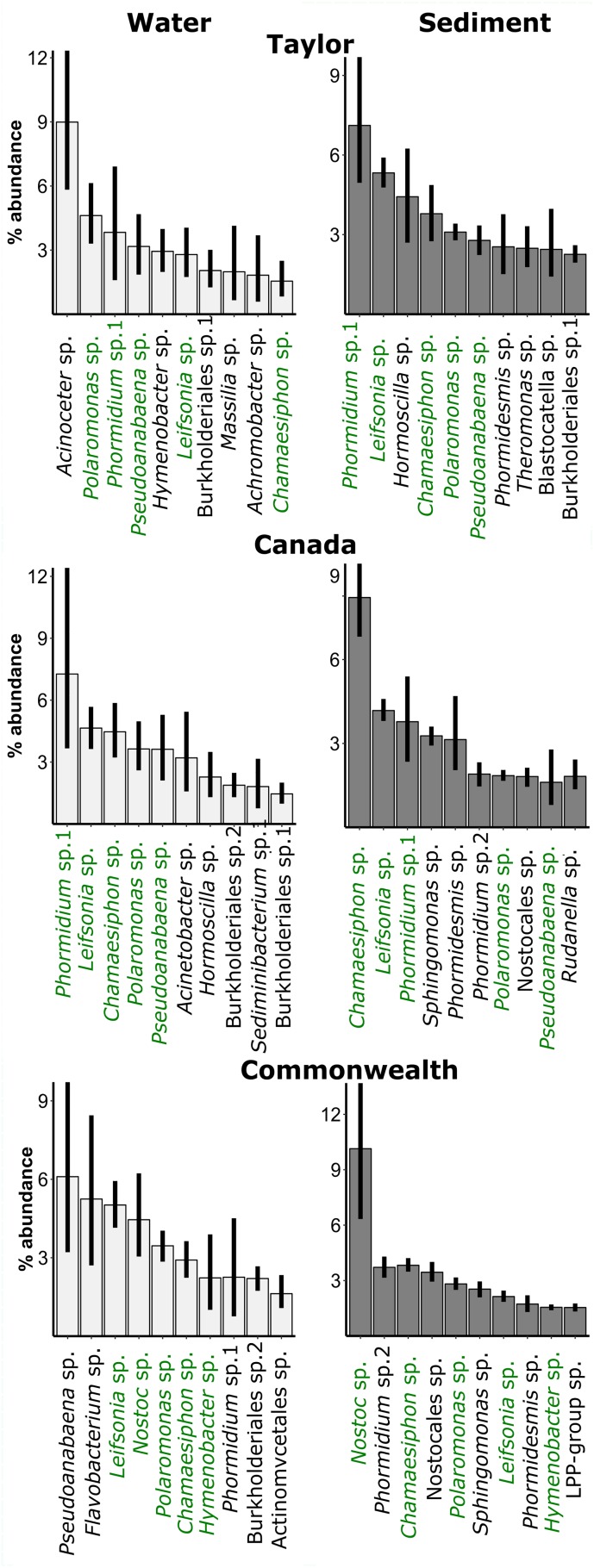
Relative abundance of the dominant bacterial OTUs in the water (left panels) and sediments (right panels) of cryoconite holes across three glaciers. Green: OTU is in the ten most abundant for both water *and* sediments on that glacier. Black: OTU is in ten most abundant of water *or* sediments on that glacier. Error bars are bootstrapped 95% confidence intervals implemented in “ggplot2” (Wickham, 2009) in R.

We furthermore plotted the ten dominant bacterial OTUs for sediments and water on each glacier (Figure 4). Sediments were dominated by cyanobacteria, with the genus of the dominant OTU differing among glaciers. In particular, the dominant cyanobacterium in Commonwealth sediments, *Nostoc* sp., was more dominant than the most dominant cyanobacterium on other glaciers, a *Chamaesiphon* sp. on Canada and a *Phormidium* sp. on Taylor. *Polaromonas* sp. (Betaproteobacteria) was also consistently in the ten most relatively abundant OTUs. These same cyanobacterial OTUs and the same *Polaromonas* sp. OTU also appeared in the water along with some other organisms, such as an *Acinetobacter* sp. (Gammaproteobacteria), that were not dominant in the sediments.

**FIGURE 5:**
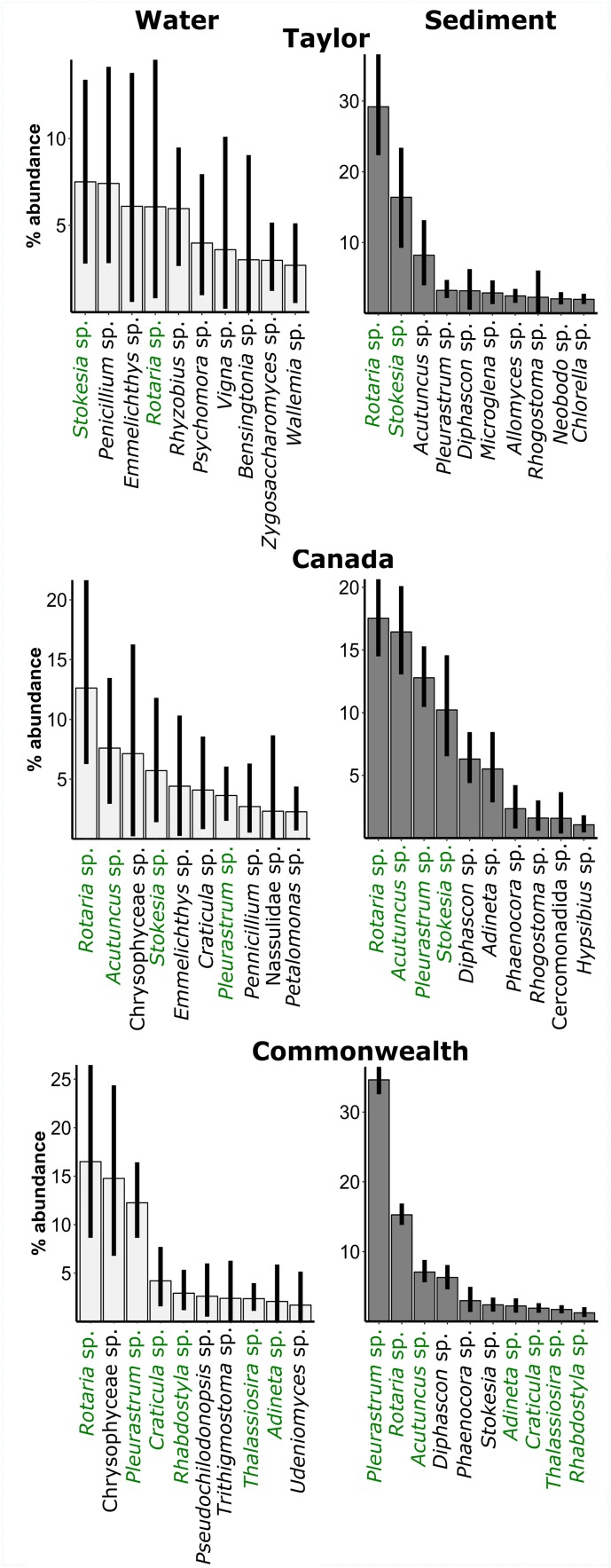
Relative abundance of the dominant eukaryotic OTUs in the water (left panels) and sediments (right panels) of cryoconite holes across three glaciers. Green: OTU is in the ten most abundant for both water *and* sediments on that glacier. Black: OTU is in ten most abundant of water *or* sediments on that glacier. Error bars are bootstrapped 95% confidence intervals implemented in “ggplot2” (Wickham, 2009) in R.

Dominant eukaryotes in the sediments included an alga OTU (*Pleurastrum* sp.), bdelloid rotifers (*Rotaria* sp. and *Adineta* sp.), tardigrades (*Acutuncus* sp. and *Diphascon* sp.), and a ciliate, *Stokesia* sp. (Figure 5). The alga was much more dominant on Commonwealth Glacier than on the other glaciers. Dominant OTUs in the water included some of those taxa, but also OTUs representing organisms that are visibly absent from cryoconite holes, such as teleost fish (*Emmelichthys* sp). Those OTUs were completely absent from the sediments, as were those of *Chrysophyceae*, despite their prevalence in the water.

### Biomass and Production

**FIGURE 6:**
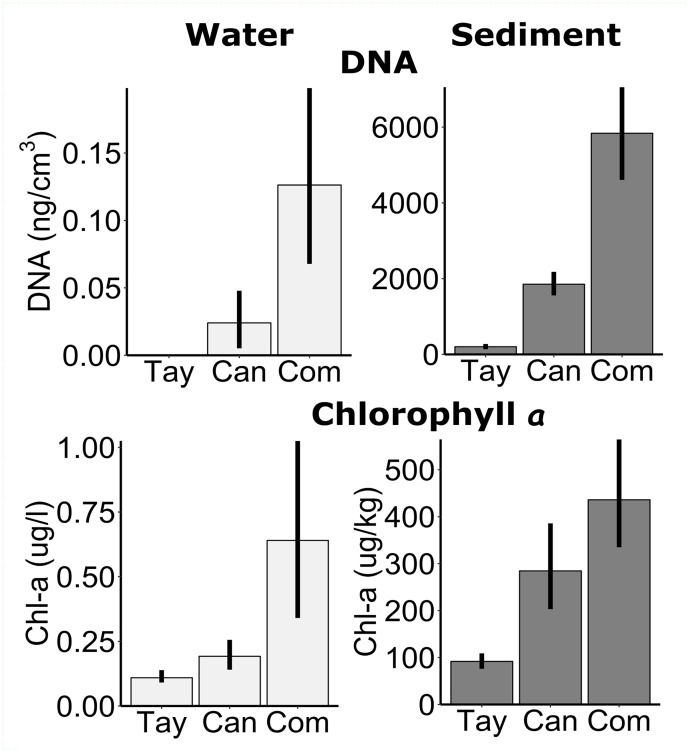
Measures of biomass proxies from the sediments and water in frozen cryoconite holes across three glaciers along a gradient of diversity. Measures include the concentrations of DNA in ng DNA/cm^3^ (top row), and chlorophyll *a* extracted per 1 l from the water and per 1 kg dry sediments (bottom row). Note the difference axis scale between water (left panels) and sediment (right panels). Tay: Taylor Glacier, Can: Canada Glacier, Com: Commonwealth Glacier. Error bars are bootstrapped 95% confidence limits, as implemented by ggplot2 (Wickham, 2009).

The proxies for biomass and primary production were substantially greater in the sediments than in the water, as expected. DNA concentrations per cm^3^ (*N*_1_ = *N*_2_ = 45) were four orders of magnitude greater in the sediments than the water (*F* = 1100, *P* < 0.001), even on Commonwealth Glacier, which had the highest concentrations (*F* = 41, *P* < 0.001) (Figure 6). Concentrations of chlorophyll *a* (*N*_1_ = *N*_2_ = 65) were also significantly higher in the sediments than in the water (*F* = 4100, *P* < 0.001), and differed among glaciers (*F* = 51, *P* < 0.001) with Commonwealth Glacier having the highest concentrations and Taylor Glacier the lowest (Figure 6).

## Discussion

As expected, OTU richness and the concentrations of DNA and chlorophyll *a* were much greater in the sediments of cryoconite holes than in the water, and the sediments and water contained distinct assemblages of bacteria and microbial eukaryotes. The richness and structure of their assemblages also differed among glaciers, in both the sediments and water, in agreement with past work (Sommers et al., 2018). However, contrary to our main hypothesis, the overlap of OTUs between sediments and water was greater on the glaciers with higher biomass proxies and OTU richness (Canada and Commonwealth) than on the glacier with lower biomass and richness (Taylor).

The greater overlap in OTUs between the sediment and water on richer glaciers is inconsistent with the idea that partitioning of sediment and water supports greater richness by increasing the niches available to support more species. Our work does not rule out that spatial partitioning may increase diversity at finer scales, both within the sediments (e.g., anaerobic microsites) and within the water (e.g., organisms attached to suspended particles). The greater biomass on Canada and Commonwealth Glaciers could have led to higher probabilities of abundant organisms being detected in both the sediments and water. The most abundant sequences from the sediments tended to be detected in the water, consistent with this explanation. Additionally, the partitioning of resources, consumers, or other factors could promote coexistence and OTU richness regardless of spatial relationships (Chesson, 2000). Finally, some abundant organisms could primarily inhabit the water while they are active during the peak of the melt season (as in Mieczan et al., 2013), but become inactive near the end of the season (as in Hawes et al., 2011; Safi et al., 2012), allowing gravity and freeze compression to settle them to the sediments. Modeling of Antarctic cryoconite holes indicate that they freeze from the top down at the end of the summer melt season (Zamora, 2018), creating the potential for a downward-moving freezing front to exclude ions and biological particles from the water (as in Watterson, 1985). This same process occurs in coastal Antarctic ponds, creating brine layers at the bottoms (Wait et al., 2006; Hawes et al., 2011; Safi et al., 2012). Such concentration in the benthic sediments, whether due to inactivity and settling or to freeze concentration, would generate a seasonal component to sediment-water partitioning in cryoconite holes. Further work on cryoconite holes should therefore investigate the seasonality of organisms’ activity in cryoconite holes, and sediment-water partitioning of the broader community during peak melt as a comparison.

Bacterial assemblages in the sediments were dominated by cyanobacteria, which are common in aquatic ecosystems in the McMurdo Dry Valleys (McKnight et al., 1998), and contribute significantly to the *in situ* primary production of the valleys (Barrett et al., 2006). This dominance is consistent with cryoconite holes having the potential to be net photosynthetic ecosystems (Bagshaw et al., 2016a), and consistent with results from other cryoconite holes in the region (Webster-Brown et al., 2015). While this was particularly true on the Commonwealth Glacier, even the lowest biomass Taylor Glacier had a diverse array of cyanobacteria. As larger Antarctic ponds freeze, their primary production slows to the point that they become net heterotrophic systems, and the relative abundance of primary producers may be at a minimum during the winter (Hawes et al., 2011; Safi et al., 2012). Assuming the same processes occur in cryoconite holes, this relative abundance of cyanobacteria in their frozen state is a conservative measure of their relative abundance. The dominant cyanobacteria in the sediments of each glacier differed, possibly due to localized Aeolian transport (Stanish et al., 2013), which was also found to be the case on other glaciers in the Dry Valleys (Webster-Brown et al., 2015). The prevalence of an OTU matching *Nostoc* sp. on the Commonwealth Glacier was particularly striking. The ability of the *Nostoc* genus to fix atmospheric nitrogen into a biologically usable form could provide an escape from nutrient limitations on glaciers (Dodds et al., 1995), and Commonwealth had the greatest chlorophyll concentrations. However, there were also some similarities among glaciers, with a *Chamaesiphon* sp. and a *Leifsonia* sp. being dominant cyanobacteria on all three glaciers. A *Polaromonas* sp. was also consistently in the ten most relatively abundant OTUs. *Polaromonas* phylotypes are regularly part of polar and alpine microbial communities globally (Darcy et al., 2011).

The eukaryotic assemblage in the sediments was primarily composed of algae, microfauna, and ciliates. The dominant alga, a *Pleurastrum* sp., falls within the “CP clade” (*Chlamydopodium-Pleurastrum*) as described by Schmidt et al. (2011). This monophyletic clade also encompasses a “snow” alga (AF514408) isolated from the high (78°N) Arctic, algae inhabiting talus soils at the Niwot Ridge Long Term Ecological Research site (Freeman et al., 2009), and algae found in the sediments on top of the debris-covered Toklat Glacier in central Alaska (e.g., KM870774) (Schmidt and Darcy, 2015). Members of the CP-clade have been found to make up a large proportion of sequences in molecular analyses of both High Himalayan soils (up 36% of 18S sequences, Schmidt et al., 2011) and some Antarctic Dry Valley soils (Fell et al., 2006). However, members of this clade have also been cultured from Lake Fryxell and other lakes in Antarctica (De Wever et al., 2009), so it is unclear if they are terrestrial algae that end up in lakes or lake algae that end up in soil/sediments. The CP clade has been previously detected in a cryoconite hole on Canada Glacier (Christner et al., 2003). Dominant microfauna included the bdelloid rotifers *Rotaria* sp. and *Adineta* sp. and the tardigrades *Acutuncus* sp. and *Diphascon* sp. It is important to keep in mind that OTUs do not translate directly to species, and that the diversity of both rotifers and tardigrades in cryoconite holes is possibly underestimated by using the V9 region of the 18S SSU gene marker (Tang et al., 2012). Sequences from a ciliate, *Stokesia* sp., and a flatworm related to *Phaenocora* sp. were also common, as previously seen in DNA from these glaciers (Sommers et al., 2018).

Dominant OTUs in the water included some of the OTUs common in the sediments, but also sequences whose closest matches were organisms that are visibly not present in cryoconite holes, such as teleost fish, *Emmelichthys* sp., and the insects *Rhyzobius* sp. (Coleoptera) and *Xylocoris* sp. (Hemiptera). These sequences could reflect the presence of microbial animals without close relatives in the SILVA 128 RefNR99 database, and that therefore match to some other animal. As they were completely absent from the richer sediments, they could also be reflective of the low DNA concentrations, and reflect lab contamination or atmospheric deposition of DNA from dead cells that became trapped in the ice. We did not detect these sequences in the biologically richer sediments, although they may have been present but not detectable given the higher biomass and concentrations of DNA. While we cannot completely rule out the possibility of contamination, their presence after filtering out OTUs from extraction blanks suggests the DNA was in the water samples and not a reflection of contamination.

Such OTUs could plausibly represent a signal of atmospheric deposition of relic DNA that remained undegraded in the low-biomass water. Wind is a major driver of biotic and abiotic processes in Taylor Valley, and plays a role in cryoconite hole formation by transporting sediments onto glaciers (Lancaster, 2002). The microbial communities of closed cryoconite hole sediments in the Taylor Valley and the surrounding McMurdo Dry Valleys show a strong signal of dispersal from local habitats, with some dispersal from farther away (Webster-Brown et al., 2015; Sommers et al., 2018) with little spatial structuring of cryoconite hole communities at the within-glacier scale (Darcy et al., 2018). Material is primarily transported down valley by powerful föhn winds (Šabacká et al., 2012), common in winter, although the gentle up-valley onshore breezes, more common in summer, could bring DNA or light microbial material from the ocean into the valley (Nylen et al., 2004). Based on these wind patterns, however, we might expect to find evidence of coastal relic DNA more common in the Commonwealth Glacier samples than those of the Taylor Glacier. The dominance of suspected inactive taxa in the holes of Taylor Glacier, therefore, may be less driven by wind patterns than its low DNA concentrations.

Some of the OTUs found only in the water may be organisms truly able to grow in cryoconite holes. For example, the *Massilia* species in the water, which was not detected in our control blanks, matched sequences from liquor fermentation (Pang et al., 2018: MG859189), but also sequences transported by coastal winds (unpubl.: MG271571 and MG270648). However, many members of its class (Burkholderiales) are also very adaptable showing high rates of horizontal gene transfer and are often early-successional taxa (Nemergut et al., 2007) that can be globally dispersed in cold environments (Darcy et al., 2011). Similarly, the closest matches for the fungus *Penicillium* sp. were from aquatic areas with human contamination, such as municipal water and water tower systems, and in canals (e.g., KT265809, KX610136, and KX090324), but *Penicillium* species have been isolated from the soils of Antarctic islands, where they were relatively abundant (Gomes et al., 2018). Another eukaryote that was nearly absent in the sediments, but dominant in the water, was a golden algae (class Chrysophyceae). Its closest relatives are from marine samples, but it is also related to the “Hydrurus clade” described by Klaveness et al. (2011). Members of this clade occur almost exclusively in high mountain or other cold environments including Baltic Sea Ice (FN690692), high-mountain snow (AJ867745), high-Arctic snow (HQ230104), and high-mountain streams (AY689714) (Klaveness et al., 2011). Unlike vertebrates, relatives of these taxa could conceivably be active in a cryoconite hole and represent an active part of the community, or they could represent contamination or relic DNA from dead cells, detectable only because of the low biomass in the frozen water.

The bulk of the DNA and chlorophyll *a* were found in the sediments of cryoconite holes across all glaciers, as in previous work on Canada Glacier’s cryoconite holes (Foreman et al., 2007). Although we expected greater biomass in the sediments than the water, similar to most aquatic systems, the degree of the difference was surprising. Previous work had found approximately four times the number of bacterial cells in the sediments compared with the water (Foreman et al., 2007), whereas we found orders of magnitude differences in the proxies of biomass we used. A previous measurement of chlorophyll *a* within melted Canada Glacier cryoconite hole water was approximately five times our measurements of the water (Foreman et al., 2007), but more mixing with the productive sediments could have taken place for the previous measurement. Differences in the extraction protocols could make DNA concentrations less comparable between sediments and water, but DNA from 50 ml in the water was mostly undetectable by fluorometry, even when concentrated, suggesting biological material was extremely low in the frozen water relative to 0.3 g sediment, potentially allowing relic or contaminant DNA or rare organisms to be detected primarily in the water. Low biomass in the water relative to the sediments is furthermore consistent with regional Antarctic ponds (Foreman et al., 2011; Hawes et al., 2011).

Pond-sized aquatic ecosystems have been proposed as good model systems for studying macroecological processes (Adrian et al., 2009; Williamson et al., 2009; Hortal et al., 2014). The response of planktonic microbial communities in Antarctic ponds to freezing has been previously characterized (Hawes et al., 2011; Safi et al., 2012), and the substantial differences in biogeochemistry factors between ponds with the same climate make them useful model systems (Archer et al., 2016). Ponds, however, are difficult to create experimentally. Antarctic cryoconite holes could serve as an even smaller study system for understanding the physical and biological interactions in lentic systems that repeatedly melt and freeze. With hundreds of holes in close proximity formed in the same glacial ice, the effect of freezing solid on the stratification of microbial life can be studied with highly replicated experiments or observational studies, which are less feasible in pond-sized ecosystems.

## Conclusion

In conclusion, we found that cryoconite holes with more OTUs and more biomass had greater overlap in OTUs present in both the sediment and water. This was contrary to a hypothesis that richer environments might be so due to partitioning of the sediments and water. Possible explanations include that other factors create niche partitioning to promote diversity, that spatial partitioning occurs at a finer scale than sampled, or that organisms primarily inhabiting the water were compressed by freezing down to the sediment layer, leaving primarily relic DNA in the frozen water to be sampled. Future work should include determining seasonal activity levels of dominant taxa in cryoconite holes, and whether changes in relative abundance are observed between the water of melted and frozen holes.

## Author Contributions

SS, PS, DP, JD, FZ, and AF determined the study design. PS, DP, JD, and FZ collected and processed the samples. FZ and AF collected and analyzed the physical data on natural cyroconite holes. PS, EG, JD, DP, SS, KC, KV, AS, LV, and JR contributed to analysis and interpretation of biogeochemical and community data. PS, EG, FZ, and AF primarily wrote the manuscript. All authors contributed text and revisions to the manuscript.

## Conflict of Interest Statement

The authors declare that the research was conducted in the absence of any commercial or financial relationships that could be construed as a potential conflict of interest.
